# Safety Assessment of *Lactiplantibacillus* (formerly *Lactobacillus*) *plantarum* Q180

**DOI:** 10.4014/jmb.2106.06066

**Published:** 2021-08-11

**Authors:** Yoo Jin Kwon, Byung Hee Chun, Hye Su Jung, Jaeryang Chu, Hyunchae Joung, Sung Yurb Park, Byoung Kook Kim, Che Ok Jeon

**Affiliations:** 1 Probiotics Research Laboratory, Chong Kun Dang Bio Research Institute (CKDBIO), Gyeonggi 15064, Republic of Korea; 2 Department of Life Science, Chung-Ang University, Seoul 06974, Republic of Korea

**Keywords:** *Lactiplantibacillus* plantarum Q180, safety, antibiotic resistance, virulence factor, probiotics

## Abstract

The safety of the probiotic strain Q180, which exerts postprandial lipid-lowering effects, was bioinformatically and phenotypically evaluated. The genome of strain Q180 was completely sequenced, and single circular chromosome of 3,197,263 bp without any plasmid was generated. Phylogenetic and related analyses using16S rRNA gene and whole-genome sequences revealed that strain Q180 is a member of *Lactiplantibacillus* (*Lp.*, formerly *Lactobacillus*) *plantarum*. Antimicrobial resistance (AMR) genes were bioinformatically analyzed using all *Lp. plantarum* genomes available in GenBank, which showed that AMR genes are present differently depending on *Lp. plantarum* strains. Bioinformatic analysis demonstrated that some mobile genetic elements such as prophages and insertion sequences were identified in the genome of strain Q180, but because they did not contain harmful genes such as AMR genes and virulence factor (VF)- and toxin-related genes, it was suggested that there is no transferability of harmful genes. The minimum inhibition concentrations of seven tested antibiotics suggested by the European Food Safety Authority guidelines were slightly lower than or equal to the microbiological cut-off values for *Lp. plantarum*. Strain Q180 did not show hemolytic and gelatinase activities and biogenic amine-producing ability. Taken together, this study demonstrated the safety of strain Q180 in terms of absence of AMR genes and VF- and toxin-related genes as a probiotic strain.

## Introduction

Probiotics are defined as “live microorganisms which when administered in adequate amounts confer a health benefit on the host” (FAO/WHO 2002). While probiotics have several known benefits, there have been safety issues related to their use in humans and animals. In 2002, the Food and Agriculture Organization/World Health Organization (FAO/WHO) reported that probiotics may cause side-effect such as systemic infection, deleterious metabolism, and excessive immune response in susceptible subjects or exhibit harmful gene transfer (FAO/WHO 2002). Furthermore, the European Food Safety Authority (EFSA) announced safety assessment guidance for probiotics by focusing on antimicrobial resistance (AMR) [1] and made it mandatory to examine susceptibility of all bacterial strains used as feed additives to the most relevant antibiotics. As a basic requirement, the minimum inhibitory concentration (MIC) should be determined for nine antibiotics (ampicillin, vancomycin, tetracycline, and others). The resistance of a bacterial strain to any specific antibiotic higher than the microbiological cut-off values defined by EFSA is deemed indicative of the presence of acquired resistance. Further, additional information is needed on the genetic basis of the AMR. For the genetic safety evaluation of microorganisms, EFSA recently recommended the taxonomic identification and characterization of their potential functional traits of concern, which may include virulence factors (VFs) and AMR [2, 3].

*Lactobacillus plantarum*, one of the lactic acid bacteria (LAB) widely used worldwide as a probiotic [4-7], was differently classified from genetically related *Lactobacillus* species on the basis of whole genome sequencing data and newly denominated as *Lactiplantibacillus* (*Lp*.) *plantarum* [8]. Many researchers have reported the safety of *Lp. plantarum* using molecular biological approaches, microbiological tools, and bioinformatics analyses [9-13]. However, the safety of *Lp. plantarum* strains cannot be guaranteed without verification, as some *Lactobacillus* strains have been reported to exhibit AMR and may not meet the EFSA guideline criteria. The most representative case was the intrinsic resistance of *Lp. plantarum* to vancomycin [14-16], caused by cell wall composition and structural changes [17]. Intrinsic resistance to antibiotics (sometimes termed as natural resistance) [1], inherent to a bacterial species, is considered relatively safe. In contrast to intrinsic resistance, non-intrinsic resistance to other antibiotics has been reported for other *Lp. plantarum* strains [18-20]. The most important aspect while dealing with AMR is non-intrinsic and acquired resistance, which can be transferred to other bacteria by horizontal gene transfer (HGT). To evaluate whether the AMR is intrinsic or acquired, we must determine the presence of specific regions of mobile genetic elements (MGEs). The genes related to VFs could also be transferred to other bacteria through the HGT mechanism. In this respect, every regulatory authority demands verification of the safety of novel probiotic strains at the genetic level to approve their commercial applicability. Therefore, it is imperative to develop systematic and definite methods at biochemical and genetic levels to assess the safety of new probiotic strains.

In our previous studies, strain Q180 isolated from the feces of a healthy Korean adult was found to exhibit blood triglyceride lowering effects in vivo and in clinical trials [21-23]. We intended to register strain Q180 as a probiotic strain with the Korea Food and Drug Administration (KFDA) and commercialize it as a probiotic product in Korea. Therefore, this study was aimed to verify the safety of strain Q180 through bioinformatic analysis and other safety tests. In this direction, we performed MIC test, hemolysis assay, biogenic amine (BA) production, and bioinformatic analyses to identify the genes related to AMR, VFs, or MGEs.

## Materials and Methods 

### Bacterial Strains and Culture Conditions

Strain Q180 was provided by the Korea Food Research Institute (KFRI, Korea) [23], and *Lp. plantarum* ATCC 14917^T^, *Enterococcus faecium* ATCC 19434^T^, and *Staphylococcus aureus* ATCC 6538 were purchased from their culture collection centers and used as reference strains for the comparison of phenotypic properties. Strains Q180, ATCC 14917^T^, and ATCC 19434^T^ were cultured in deMan-Rogosa-Sharpe (MRS; BD, USA) broth, and strain ATCC 6538 was cultured in tryptic soy broth (BD) at 37°C. The bacterial cultures were mixed with 20% skim milk (BD) at a 1:1 ratio and preserved at −80°C. All test experiments were performed using freshly cultured cells from frozen stocks and not from consecutively subcultured cells.

### Whole Genome Sequencing of Strain Q180

The genomic DNA of strain Q180 was extracted and completely sequenced using the combination of PacBio RS II and Illumina HiSeq 2500 platforms at Macrogen (Korea), as previously described [24]. In brief, the genomic DNA of strain Q180 was sequenced using PacBio RS II with a 10 kb library, and the resulting sequencing reads were *de novo* assembled using the Hierarchical Genome Assembly Process software (ver. 3.0) and the PBcR pipeline of Celera Assembler [25]. The completely assembled genome derived from the PacBio sequencing data was error-corrected by Illumina sequencing reads using the Pilon software (ver. 1.21) [26].

### Phylogenetic and Genome-Related Analyses

Taxonomic identification of strain Q180 was conducted through phylogenetic analyses based on the 16S rRNA gene and whole-genome sequences. For the 16S rRNA gene sequence-based phylogenetic analysis, the 16S rRNA gene sequences of strain Q180 and closely related type strains were aligned using the fast secondary-structure-aware infernal aligner available in the ribosomal database project [27]. A maximum-likelihood (ML) tree with bootstrap values (1,000 replications) was constructed using the MEGA7 software [28]. The 16S rRNA gene sequence similarities between strain Q180 and closely related type strains were calculated using the EzTaxon-e server (http://www.ezbiocloud.net/). For the genome-based phylogenetic analysis, 92 housekeeping core genes from the genomes of strain Q180 and closely related type strains were extracted using the UBCG pipeline (www.ezbiocloud.net/tools/ubcg) [29], and an ML tree with bootstrap values (1,000 replications) based on the concatenated nucleotide sequences of the 92 housekeeping core genes was constructed using the MEGA7 software.

The genome relatedness between strain Q180 and closely related type strains was evaluated through average nucleotide identity (ANI) and digital DNA-DNA hybridization (DDH) analyses using the Orthologous Average Nucleotide Identity Tool software (www.ezbiocloud.net/sw/oat) [30] and the server-based Genome-to-Genome Distance Calculator (http://ggdc.dsmz.de/distcalc2.php) [31], respectively.

### Genomic Analysis

The circular map of the assembled genome of strain Q180 was visualized using a web-based CGview program [32]. The complete genome of strain Q180 was submitted to GenBank for the gene prediction and functional annotation. Protein-coding sequences in the genomes of strain Q180 and *Lp. plantarum* DSM 20174^T^ were functionally classified into clusters of orthologous groups (COG) categories using eggNOG-mapper (ver. 5.0)[33].

### Bioinformatic Analysis of AMR Genes, VF- and Toxin-Related Genes, and MGEs

AMR genes present in the genomes of all *Lp. plantarum* strains, including strain Q180 (CP073753) and *Lp. plantarum* DSM 20174^T^ (CP039121), available in GenBank were analyzed. All genomes of *Lp. plantarum* were downloaded from the GenBank database, and AMR genes in each genome were searched through BlastX analysis using Diamond software (ver. 0.9.26.127) [34] based on Comprehensive Antibiotic Resistance Database (CARD)[35], Antibiotic Resistance Gene-ANNOTation (ARG-ANNOT) [36], and ResFinder databases [37]. The functional properties of the searched AMR genes were confirmed through BlastX analysis against the NCBI nr and UniRef90 databases.

VF- and toxin-related genes, including those associated with enterotoxin, leucotoxin, cytolysin, cytotoxin K, hemolysis, BA production, hyaluronidase, aggregation, enterococcal surface protein, endocarditis antigen, collagen adhesion, cereulide, sex pheromone, and serine protease were searched in the genomes of strain Q180, *Lp. plantarum* DSM 20174^T^, *E. faecium* ATCC 19434^T^ (UFYJ00000000), and *S. aureus* ATCC 6538 (CP020020–21) based on the virulence factor database (VFDB) using BlastX available in the Diamond software [38]. These genes were additionally confirmed through BlastX analysis using experimentally verified VF and toxin genes in the UniRef90 database.

To assess the transferability of AMR/VF genes in strain Q180, prophages and insertion sequences (including transposons), which are representative MGEs, were investigated using PHAge Search Tool Enhanced Release (PHASTER) [39] and ISfinder [40], respectively.

### Prophage Induction Test in Strain Q180

The inducibility of the putative prophage sequences as active phages in strain Q180 was evaluated using a mitomycin C approach, as described previously [41]. In brief, strain Q180 was cultured to an optical density of 0.2 (600 nm) at 37°C in 100 ml MRS broth supplemented with 10 mM CaCl_2_ and mitomycin C was added to be a final concentration of 0.6 μg/ml, followed by additional incubation at 30°C for 18 h. Phage DNA was isolated from the cell culture supernatants, according to the phage DNA extraction protocol described previously [42], and detected on 1.0% (w/v) agarose gel.

### Determination of MICs of Antibiotics

Phenotypic resistance of strain Q180 and *Lp. plantarum* ATCC 14917^T^ for seven antibiotics (ampicillin, gentamicin, kanamycin, erythromycin, clindamycin, tetracycline, and chloramphenicol) was assessed by measuring the MIC of each antibiotic using the E-test strip and broth microdilution susceptibility assay methods. The MIC tests were performed in triplicates for each antibiotic. For the MIC assay using E-test strip, bacterial suspensions were spread onto agar media containing LAB susceptibility medium (LSM, 90% IsoSensitest broth [Oxoid, UK], 10% MRS broth) supplemented with 1.5% agar (BD). E-test strips (Liofilchem, USA) were applied to the center of each agar plate, which was incubated for 24 h at 37°C. The lowest concentrations of antibiotics that inhibited the visible growth of strain Q180 and *Lp. plantarum* ATCC 14917^T^ on LSM agar were determined as MICs. The MICs of each antibiotic for strain Q180 and *Lp. plantarum* ATCC 14917^T^ were also assessed according to the standard broth microdilution susceptibility assay protocol recommended by the National Committee for Clinical Laboratory Standards [43].

### Phenotypic Tests for Hemolytic and Gelatinase Activities and BA-producing Ability

Phenotypic tests for hemolytic and gelatinase activities of strain Q180 were performed, and BA-producing ability of strain Q180 was investigated. *Lp. plantarum* ATCC 14917^T^, *E. faecium* ATCC 19434^T^, and *S. aureus* ATCC 6538 were used as negative or positive controls. Frozen stocks of bacterial strains were streaked on MRS agar and incubated at 37°C for 24 h. Single colonies grown on MRS agar were inoculated into MRS broth, and an aliquot of the cultured broth was used for the tests. All experiments were performed in triplicates.

To evaluate hemolytic activity, each strain was streaked on blood agar containing 5% (w/v) sheep blood (MB Cell, Korea) and the plates were incubated at 37°C for 48 h. The hemolytic activity (*α*, *β*, and *γ*) of the strains was evaluated based on the formation of hemolysis zones around colonies grown on blood agar [44] To test gelatinase activity, each strain was inoculated on a gelatin medium (120 g gelatin, 0.5 g peptone, and 0.3 g beef extract per liter) by stabbing an aliquot from the cultured broth and incubating the plates at 37°C for 4 days. The agar plates were placed at 4°C for 10 min to observe gelatin degradation by gelatinase activity.

The BA-producing ability of each strain was assessed according to a previously described procedure [45]. In brief, the test strains were cultivated in MRS broth containing histidine, tyrosine, ornithine, and lysine (each 0.25%) at 37°C for 2 days. The culture broths were syringe-filtered (0.2 μm; Biofact, Korea). For 9-fluorenylmethoxy carbonyl (FMOC) derivatization, 20 μl of the filtrates or standards (5, 10, and 20 μM of histamine, tyramine, putrescine, and cadaverine; Sigma-Aldrich, USA) and 200 μl of 1.5 mM FMOC (in acetone) were added to 200 μl of 0.5 M sodium borate buffer (pH 8.5) containing 20 μM norvaline (internal standard) and vigorously mixed. After 3 min incubation at room temperature (dark), 50 μl of 10 mM glycine in 0.5 M sodium borate buffer was added to the mixture to remove excess FMOC. FMOC-derivatized BA was analyzed by high-performance liquid chromatography (HPLC; Shimadzu, Japan) equipped with a reverse-phase C_18_ column (250 × 4.6 mm) and a fluorescence detector (RF-10AXL) as described by Brückner *et al*. [46].

## Results and Discussion

### Genomic Sequencing and Taxonomic Identification of Strain Q180

**Fig. 1:**
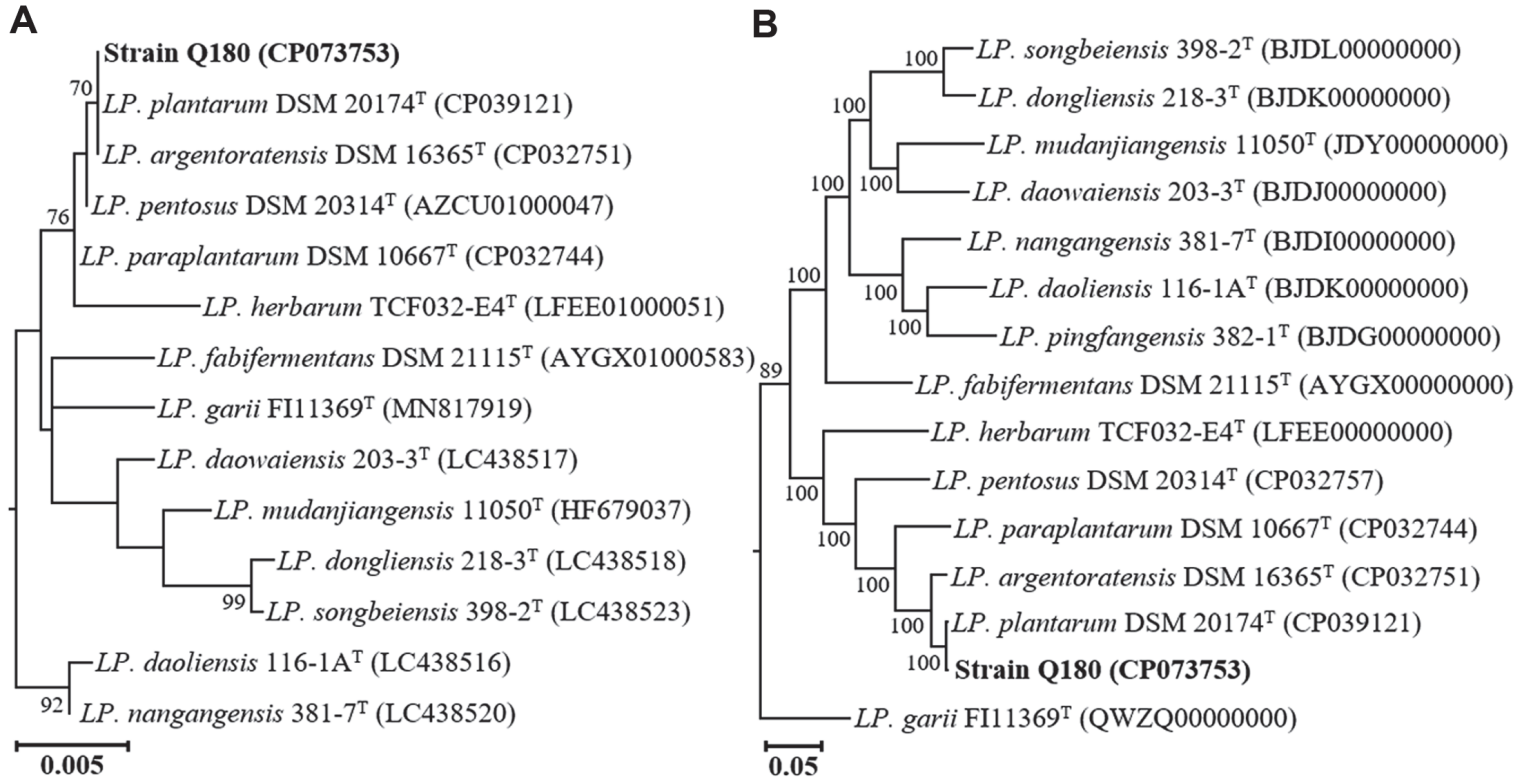
Maximum-likelihood phylogenetic trees showing the relationships between strain Q180 and the closely related *Lactiplantibacillus* type strains, based on the 16S rRNA gene (**A**) and concatenated 92 housekeeping core gene (**B**) sequences. Levilactobacillus tongjiangensis LMG 26013^T^ (JQCL00000000) was used as the outgroup (not shown). The bars, 0.005 and 0.05, represent changes per nucleotide.

**Fig. 2:**
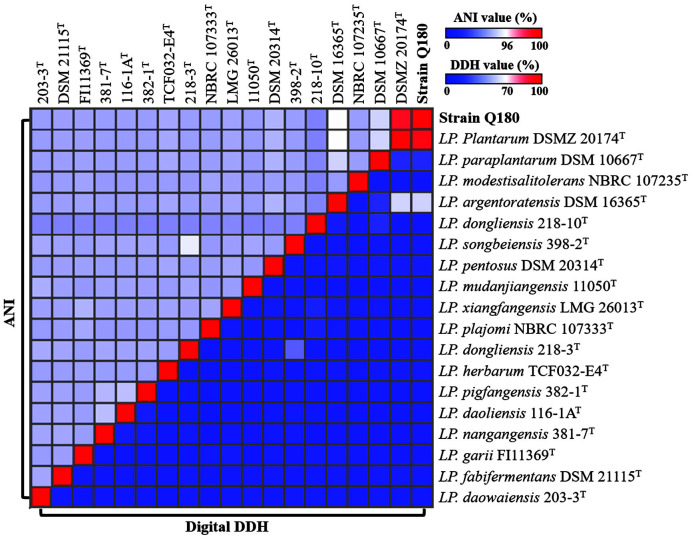
Heat-maps showing pair-wise average nucleotide identity (ANI) and digital DNA-DNA hybridization (DDH) values of strain Q180 and the closely related *Lactiplantibacillus* type strains.

The genome of strain Q180 was completely sequenced with high quality and taxonomically identified through 16S rRNA gene- and whole genome-based phylogenetic analyses. Phylogenetic analysis based on 16S rRNA gene sequences revealed that strain Q180 formed a phylogenic lineage with both *Lp. plantarum* DSM 20174^T^ and Lp. argentoratensis DSM 16365^T^ within the genus *Lactiplantibacillus* (Fig. 1A), and showed 100% similarity in 16S rRNA gene sequences. However, genome-based phylogenetic analysis using 92 housekeeping core genes showed that strain Q180 formed a phylogenic lineage with *Lp. plantarum* DSM 20174^T^ but not with Lp. argentoratensis DSM 16365^T^ (Fig. 1B), suggesting that strain Q180 may be a member of *Lp. plantarum*. Analysis of ANI and digital DDH values between strain Q180 and the closely related *Lactiplantibacillus* type strains showed that strain Q180 shared 99.2% ANI and 93.8% digital DDH values with *Lp. plantarum* DSM 20174^T^ (Fig. 2), which were clearly higher than the thresholds (ANI, ~95%; digital DDH, 70%) for the delineation of prokaryotic species [31, 47], suggesting that strain Q180 is a strain of *Lp. plantarum*. However, strain Q180 and *Lp. plantarum* DSM 20174^T^ shared lower digital DDH values (63.0% and 63.3%, respectively) with Lp. argentoratensis DSM 16365^T^ than the thresholds, suggesting that they are distinct from Lp. argentoratensis at the species level but exhibit 100% similarity in 16S rRNA gene sequences.

### Genomic Features of Strain Q180

**Fig. 3:**
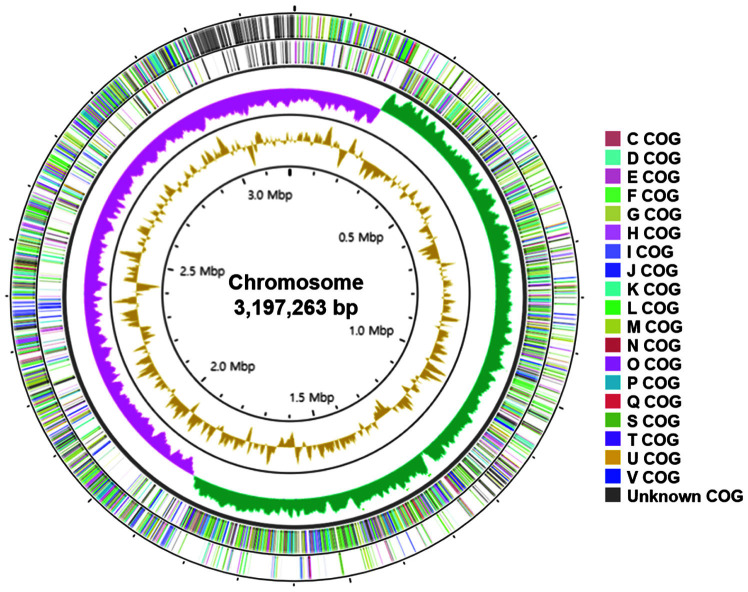
A circular map representing the chromosome of strain Q 180. Forward strand and reverse strand coding sequences on the outermost two circles of the map are differently colored according to the COG categories of the right side. GC skews (GC skew+: green, GC skew-: pink) and G+C content (yellow) are drawn on the third and fourth circles, respectively.

The key genomic features of strain Q180, including GC skew, protein coding sequences (CDSs), COG categories, and G+C contents, are graphically depicted in Fig. 3. The general genomic features of strain Q180 were summarized and compared with those of the type strain of *Lp. plantarum* (DSM 20174^T^) (Table 1). The genome of strain Q180 was a single circular chromosome of 3,197,263 bp without any plasmid, whereas *Lp. plantarum* DSM 20174^T^ had a single circular chromosome of 3,242,936 bp with a plasmid of 7,218 bp. The G+C content of the genomic DNA was 44.6 mol% for strain Q180, which had 3,301 total genes, 3,049 CDS, 16 rRNA genes, and 68 tRNA genes encoding 20 amino acids. The general features were similar to those of *Lp. plantarum* DSM 20174^T^. The number of COG-assigned proteins in the genomes of strain Q180 and *Lp. plantarum* DSM 20174^T^, and their distributions into COG categories were also similar (Table S1).

### Bioinformatic Analysis of AMR Genes and VF- and Toxin-Related Genes

During the evaluation of the safety of probiotic strains, their phenotypic properties related to AMR are considered important (WHO 2019). As the genomic analysis of microbes provides information on AMR genes as well as their potential transferability, genomic analysis is necessary for the evaluation of the potential risks of new probiotic strains. Previous genomic analysis studies have shown that many *Lactobacillus* strains harbor AMR genes that are involved in mediating resistance to several antibiotics, including β-lactam, macrolide, aminoglycoside, chloramphenicol, and tetracycline [14, 48-50]. In particular, tetracycline resistance genes such as *tetW*, *tetM*, and *tetO* have been frequently identified in *Lactobacillus* species [50, 51]. In the safety evaluation, probiotic strains were investigated at the species level, and *Lp. plantarum* strains that are widely used as probiotics were often deemed as safe. However, several studies have reported that safety issues such as AMR, virulence, and toxin-producing ability vary depending on the strain even within the same species [52, 53], suggesting that the safety evaluation of new probiotic candidate strains should be carried out at the strain level. To investigate the distribution of AMR genes in *Lp. plantarum* strains, AMR genes in all *Lp. plantarum* genomes available in GenBank were bioinformatically analyzed based on the CARD, ARG-ANNOT, and ResFinder databases (Table 2). Bioinformatic analyses showed that genes encoding antibiotic inactivation enzymes, antibiotic target protection proteins, and antibiotic efflux pumps, which may confer resistance to antibiotics such as tetracycline, lincosamide, carbapenem, and aminoglycoside, were identified in some *Lp. plantarum* strains. Thus, the presence of AMR genes may vary depending on *Lp. plantarum* strains; therefore, safety evaluation of *Lp. plantarum* strains should be carried out at the strain level. However, no AMR gene was identified in the genomes of strain Q180 and *Lp. plantarum* DSM 20174^T^ (data not shown), which suggests that strain Q180 and *Lp. plantarum* DSM 20174^T^ are considered safe as probiotics in the AMR gene issues.

The VF- and toxin-related genes were bioinformatically analyzed in the genomes of strain Q180, *Lp. plantarum* DSM 20174^T^, *E. faecium* ATCC 19434^T^, and *S. aureus* ATCC 6538 (Table 3). No VF - or toxin-related genes were identified in the genomes of strain Q180 and *Lp. plantarum* DSM 20174^T^, suggesting that strain Q180 may be safe as a probiotic strain. However, some VF- or toxin-related genes were identified in the genomes of *E. faecium* ATCC 19434^T^ and *S. aureus* ATCC 6538 known as pathogenic strains. For example, *E. faecium* ATCC 19434^T^ harbors a gene encoding tyrosine decarboxylase, suggestive of its ability to produce tyramine from tyrosine. *S. aureus* ATCC 6538 also harbors genes encoding enterotoxin, leucotoxin, and hemolysin, indicative of its ability to produce toxins and exhibit hemolytic activity.

**Fig. 4:**
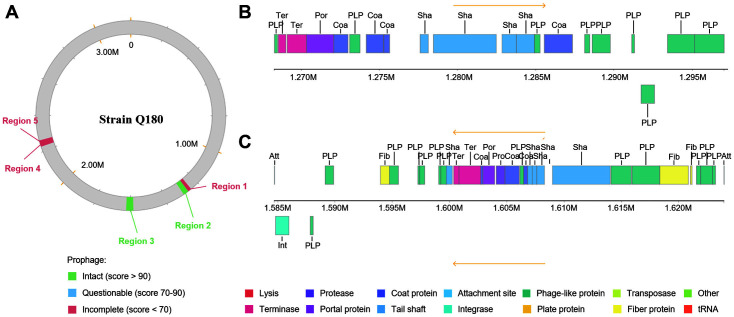
Bioinformatic analysis of phage sequences in the genome of strain Q180. A, the locations of putative prophage regions identified in the chromosome of strain Q180; B and C, the genomic maps of putative prophage regions 2 and 3 in panel A. The phage sequences were analyzed using PHASTER.

To assess the transferability of AMR/VF or toxin genes in strain Q180, the presence of MGEs, including prophages or insertion sequences, was bioinformatically investigated. Two putative intact prophages (regions 2 and 3) and three incomplete phage sequences were identified in the genome of strain Q180 (Fig. 4A), suggesting the possibility of active lateral gene transfer by phage infection. However, the prophage sequence of region 2 did not have an integrase protein, a key protein for prophage induction (Fig. 4B). Although some key proteins for prophage induction were identified from the prophage sequence of region 3, the sequences were more similar to bacterial protein-coding genes than viral genes. In-vitro induction test of the prophages also showed that the prophages were not induced (data not shown). These results suggest that the prophage sequences identified in the genome of strain Q180 may be not inducible and strain Q180 is safe from the transferability of AMR/VF or toxin genes by a prophage.

The bioinformatic analysis also showed that two types of insertion sequences (IS1182 and ISL3) annotated as transposases probably originating from *Lp. plantarum* were identified in the genome of strain Q180 (Table S2). As no AMR/VF or toxin sequences were identified from the prophage, phage, and IS element regions, and strain Q180 did not carry any plasmid (unlike *Lp. plantarum* DSM 20174^T^), it may have low transferability of AMR/VF or toxin genes via MGEs.

### Antibiotic Susceptibility Assay

Although most strains, including *Lactiplantibacillus*, classified as formerly *Lactobacillus* [8] are known to be susceptible to antibiotics, some *Lactiplantibacillus* strains have been shown to exhibit AMR [54]. It is well known that *Lp. plantarum* strains commonly exhibit intrinsic resistance to vancomycin [14] and streptomycin [18, 55, 56]. Such intrinsic AMR is considered relatively safe, owing to low transferability by HGT [1]. In addition to the intrinsic resistance of *Lp. plantarum* strains to vancomycin and streptomycin, non-intrinsic resistance to other antibiotics has been reported in some *Lp. plantarum* strains [18-20]. The bioinformatic analysis of AMR genes in *Lp. plantarum* genomes also showed that some *Lp. plantarum* strains harbor AMR genes, which are different depending on strains (Table 2), which suggests that *Lp. plantarum* strains can differ their antibiotic resistance.

The antibiotic resistance assays using the E-test strip and broth microdilution susceptibility assay methods showed that the MIC values of the seven antibiotics suggested by the EFSA guideline for strain Q180 were similar to those of *Lp. plantarum* ATCC 14917^T^ (Table 4), consistent with the bioinformatic results that no AMR gene was identified from the genomes of strain Q180 and *Lp. plantarum* ATCC 14917^T^ (Table 2). The MIC values were slightly lower than or equal to the microbiological cut-off values in the EFSA guideline for *Lp. plantarum*, suggesting that strain Q180 is susceptible to antibiotics and is acceptable as a probiotic strain.

### Hemolytic and Gelatinase Activities and BA-Producing Ability

**Fig. 5:**
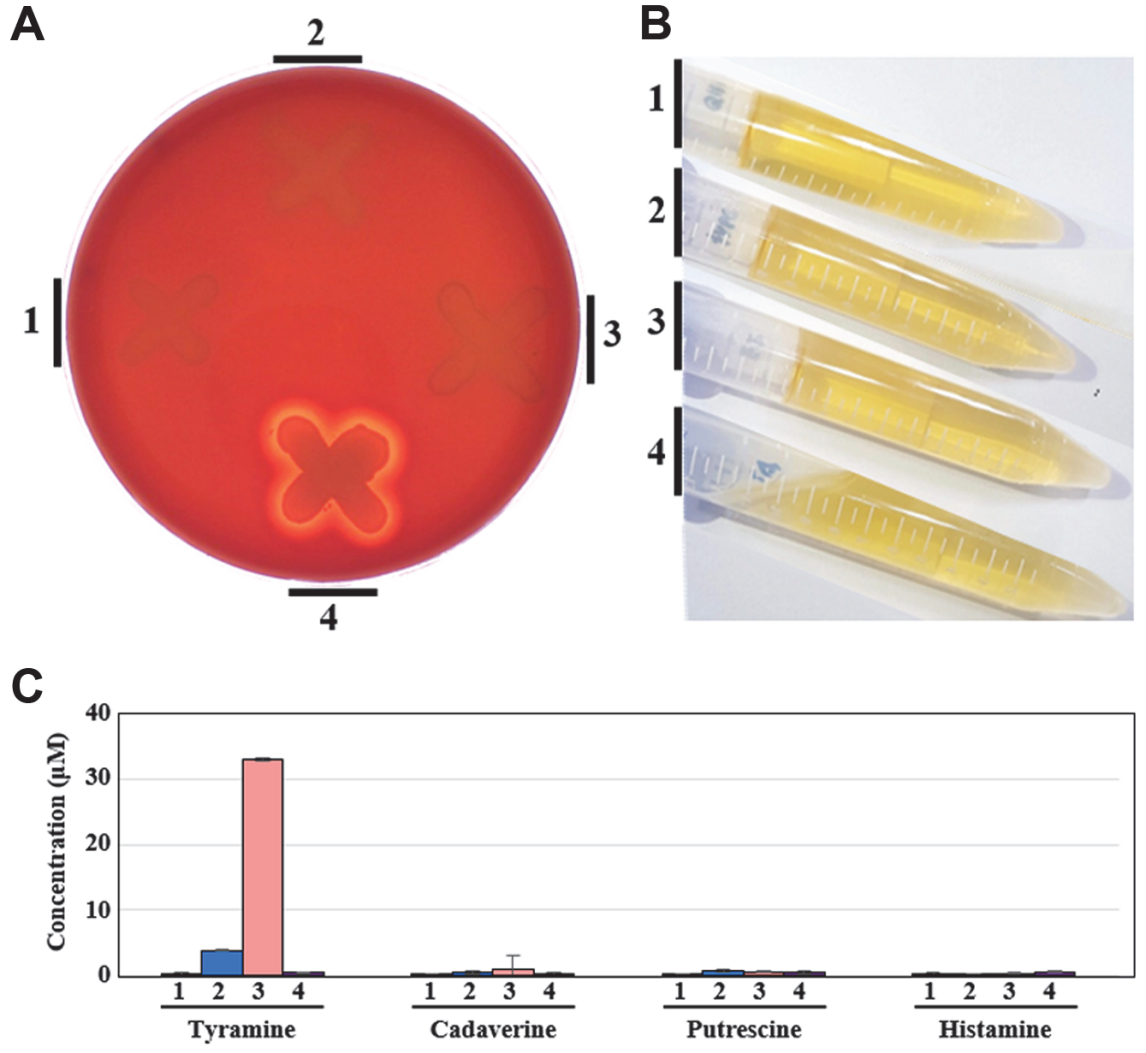
Phenotypic tests. A, hemolysis test; B, gelatinase activity; and C, biogenic amine production of 1, strain Q180; 2, *Lp. plantarum* ATCC 14917^T^; 3, *E. faecium* ATCC 19434^T^; and 4, *S. aureus* ATCC 6538.

To confirm the safety of a probiotic strain, it is necessary to phenotypically verify its virulence or toxin-related properties through in vitro tests. In this study, the hemolytic and gelatinase activities and BA-producing ability of strain Q180 were tested, together with *Lp. plantarum* DSM 20174^T^, *E. faecium* ATCC 19434^T^, and *S. aureus* ATCC 6538 as negative or positive strains, (Fig. 5). Hemolysis is the lysis of red blood cells and the release of their cytoplasmic fluids by the action of hemolysins [44]. Some pathogenic microbes, such as *S. aureus*, are known as hemolysis-causing agents [57]. Strain Q180 had no hemolytic activity (called *γ*-hemolysis), similar to *Lp. plantarum* DSM 20174^T^ and *E. faecium* ATCC 19434^T^, while *S. aureus* ATCC 6538 exhibited clear hemolytic activity (β-hemolysis) (Fig. 5A). This observation was in line with the result of bioinformatic analysis of hemolysin genes in the genomes of test strains (Table 3).

Gelatinase is a hydrophobic metalloprotease that cleaves insulin, casein, hemoglobin, collagen, and gelatin. Because it has been reported that pathogenic bacteria such as enterococci responsible for endocarditis and bacteremia have the ability to penetrate the tissue through their gelatinase activities [58], gelatinase is considered as one of the VFs. In the present study, no gelatinase activity was detected for strain Q180 as well as *Lp. plantarum* DSM 20174^T^ and *E. faecium* ATCC 19434^T^, while *S. aureus* ATCC 6538 showed clear gelatinase activity (Fig. 5B). This result was in consistent that of the bioinformatic analysis of gelatinase genes in the genomes of test strains (Table 3). Previous studies have also reported that *Lp. plantarum* strains showed no gelatinase activity [59, 60].

BAs such as histamine, tyramine, cadaverine, and putrescine, mainly formed by the decarboxylation of their corresponding amino acids, act as neurotransmitters in organisms; however, excessive intake of BAs can cause diseases or disorders such as diarrhea, vomiting, sweating, and tachycardia [61]. Here, we found that strain Q180 had no BA-producing ability, as observed for *Lp. plantarum* DSM 20174^T^ and *S. aureus* ATCC 6538 and that *E. faecium* ATCC 19434^T^ had the ability to produce tyramine (Fig. 5C). This observation was consistent with the result of bioinformatic analysis of BA-producing genes in the genomes of test strains (Table 3). Recent studies have also reported that *Lp. plantarum* strains do not have the ability to produce BAs [62-64]. Taken together, our in vitro tests showed that strain Q180 had no hemolysis and gelatinase activities and BA-producing ability, suggesting that its application as a safe probiotic owing to the absence of virulence- and toxin-related genes.

In this study, the safety of strain Q180 known to exhibit postprandial lipid-lowering effect was evaluated through bioinformatic and phenotypic analyses to test its potential as a probiotic strain. First, strain Q180 was subjected to phylogenetic and genome-related analyses, which showed that it belongs to *Lp. plantarum* that has been widely used as probiotics. Second, the whole-genome of strain Q180 was bioinformatically analyzed, and strain Q180 did not harbor potentially harmful genes such as AMR genes and VF- and toxin-related genes. Third, phenotypic tests for the presence of some virulence properties were performed, and strain Q180 was found to exhibit no AMR, hemolytic and gelatinase activities, and BA-producing ability. In conclusion, strain Q180 may be safe for human health and can be used as a potential probiotic strain to ameliorate high blood triglyceride levels. Although bioinformatic and phenotypic analyses conducted in this study can provide the basic safety information for probiotic strains, further systematic and acceptable approaches that evaluate the safety of probiotic strains should be conducted to confirm their safe and effective applications.

## Supplemental Materials

Supplementary data for this paper are available on-line only at http://jmb.or.kr.

## Figures and Tables

**Table 1:** General features of the genomes of strain Q180 and *Lp. plantarum* DSM 20174^T^.

Feature	Q180	DSM 20174^T^
No. of contigs	1	2
Chromosome size (bp)	3,197,263	3,242,936
Plasmid size (bp)	–	7,218
GC content (%)	44.6	44.5
Total genes	3,301	3,060
Protein coding genes	3,049	2,921
rRNA genes	16	16
tRNA genes	68	71
Pseudogenes	168	48
Proteins with function prediction	2,373	2,518
Proteins assigned to COG	2,526	2,525
GenBank acc. no.	CP073753	CP039121

**Table 2:** The distribution of antimicrobial resistance (AMR) genes in the genomes of *Lactiplantibacillus plantarum* strains.

ARO category	Target antibiotics	Antibiotic gene database^†^								

		CARD			ARG-ANNOT			ResFinder		
		
		P	S	L	P	S	L	P	S	L
Antibiotic inactivation enzyme	Carbapenem, aminoglycoside, phenicol antibiotic, lincosamide, and streptogramin	14	1	4	11	2	1	2	1	0
Antibiotic target protection protein	Tetracycline and lincosamide	0	1	4	1	0	7	1	0	0
Antibiotic efflux pump	Tetracycline, aminoglycoside, fluoroquinolone, fosfomycin, and macrolide	0	0	66	0	0	16	0	0	0

ARO, antibiotic resistance ontology

^†^The AMR genes were searched in a total of 583 *Lp. plantarum* genomes retrieved from GenBank based on the CARD, ARGANNOT, and ResFinder databases. The results represent the number of genomes containing at least one or more AMR genes. P, perfect hits: 100% similarity sequences with the database sequences; S, strict hits: 90%–100% similarity sequences with the database sequences; L, loose hit: 50%–90% similarity sequences with the database sequences.

**Table 3:** Bioinformatic analysis for the presence of putative virulence factor- and toxin-related genes in the genomes of strain Q180, *Lp. plantarum* DSM 20174^T^, *E. faecium* ATCC 19434^T^, and *S. aureus* ATCC 6538.

Class	Gene	*Lp. plantarum*		*E. faecium*	*S. aureus*

		Q180	DSM 20174^T^	ATCC 19434^T^	ATCC 6538
Enterotoxin	*selk, selq, set*	–	–	–	+
Leucotoxin	*lukD*	–	–	–	+
Cytolysin	*cylA*	–	–	–	–
Cytotoxin K	*cytK*	–	–	–	–
Hemolysin	*hbl*	–	–	–	+
Gelatinase	*gelE*	–	–	–	+
Amino acid decarboxylase	*hdc1*, *hdc2*	–	–	–	–
	*tdc*	–	–	+	–
	*odc*	–	–		–
	*ldc*	–	–	–	–
Hyaluronidase	*hyl*	–	–	+	+
Aggregation substance	*asa1*	–	–		–
Enterococcal surface protein	*esp*	–	–	–	–
Endocarditis antigen	*efaA*	–	–	–	–
Adhesion of collagen	*ace*	–	–	–	–
Cereulide	*cesA*	–	–	–	–
Sex pheromones	*ccf*, *cob*, *cpd*	–	–	–	–
Serine protease	*sprE*	–	–	–	+
Transposon-related genes	*int*, *intTN*	–	–	–	–

**Table 4:** Minimum inhibitory concentrations (MICs) of antibiotics for strain Q180 and *Lp. plantarum* ATCC 14917^T^.

Antibiotic	MIC (mg/l)				

	Strain Q180		Strain ATCC 14917^T^		Cut-off value^‡^

	E-test strip	Broth assay^†^	E-test strip	Broth assay^†^	
Ampicillin	1.0±0.0	0.25±0.0	0.38±0.0	0.5±0.0	2.0
Gentamicin	1.5±0.0	8.0±0.0	1.0±0.0	8.0±0.0	16.0
Kanamycin	24.0±0.0	64.0±0.0	24.0±0.0	64.0±0.0	64.0
Erythromycin	0.25±0.0	0.4±0.1	0.25±0.0	1.0±0.0	1.0
Clindamycin	0.25±0.0	0.125±0.0	0.19±0.0	0.125±0.0	2.0
Tetracycline	32.0±0.0	32.0±0.0	24.0±0.0	32.0±0.0	32.0
Chloramphenicol	8.0±0.0	8.0±0.0	6.0±0.0	8.0±0.0	8.0

^†^The assay was performed using the standard broth microdilution susceptibility assay protocol recommended by the National Committee for Clinical Laboratory Standards [43].

^‡^Microbiological cut-off values for antibiotics for *Lp. plantarum*, as provided by the EFSA 2012 guideline.
