# Supplementary material for: Safety Assessment of Lactiplantibacillus (formerly Lactobacillus) plantarum Q180
Source: J Microbiol Biotechnol. 2021 Aug 11;31(10):1420–9. doi: 10.4014/jmb.2106.06066 (PMC9705903; doi:10.4014/jmb.2106.06066)
Supplement: Supplementary file 1 [file jmb-31-10-1420-supple.pdf]

J. Microbiol. Biotechnol.
https://doi.org/10.4014/jmb.2106.06066

Safety assessment of *Lactiplantibacillus* (formerly *Lactobacillus*) *plantarum* Q180

Yoo Jin Kwon[1,†], Byung Hee Chun[2,†], Hye Su Jung[2], Jaeryang Chu[1], Hyunchae Joung[1], Sung Yurb Park[1], Byoung Kook Kim[1], and Che Ok Jeon[2,*]

[1]*Probiotics Research Laboratory, Chong Kun Dang Bio Research Institute (CKDBIO), Gyeonggi 15064, Republic of Korea*

[2]*Department of Life Science, Chung-Ang University, Seoul 06974, Republic of Korea*

[*] Corresponding author: cojeon@cau.ac.kr

Table S1. Numbers of genes classified into COG functional categories in the genomes of strain Q180 and *Lp. plantarum* DSM 20174^T

COG category	Number of genes	
	Q180	DSM 20174^T
Energy production and conversion (C)	115	114
Cell cycle control and mitosis (D)	39	38
Amino Acid metabolism and transport (E)	222	219
Nucleotide metabolism and transport (F)	127	127
Carbohydrate metabolism and transport (G)	251	266
Coenzyme metabolism (H)	95	91
Translation (J)	63	62
Lipid metabolism (I)	169	175
Transcription (K)	293	297
Replication and repair (L)	146	137
Cell wall/membrane/envelop biogenesis (M)	161	172
Cell motility (N)	15	15
Post-translational modification, protein turnover, chaperone functions (O)	55	54
Inorganic ion transport and metabolism (P)	159	158
Secondary Structure (Q)	28	28
Signal Transduction (T)	68	72
Intracellular trafficking and secretion (U)	72	72
Defense mechanism (V)	61	58
Function Unknown (S)	570	561

Table S2. List of insertion sequences identified in the genome of strain Q180 and *Lp. plantarum* DSM 20174^T

IS Family	Function	Origin	Q180		DSM 20174^T	
			Start	End	Start	End
IS1182	Transposase	*Lp. plantarum*	674,312	676,094	1,363,542	1,365,337
			–	–	88,486	90,279
			–	–	347,584	349,379
			–	–	2,834,825	2,836,620
			–	–	316,095	317,890
			–	–	1,998,610	2,000,405
ISL3	Transposase	*Lp. plantarum*	1,697,899	1,699,331	1,995,311	1,996,743
			2,892,750	2,894,182	90,281	91,359
			2,429,961	2,431,074	–	–
			264,136	264,897	–	–
